# Supplementary material for: The oldest magnetic record in our solar system identified using nanometric imaging and numerical modeling
Source: Nat Commun. 2018 Mar 21;9:1173. doi: 10.1038/s41467-018-03613-1 (PMC5862876; doi:10.1038/s41467-018-03613-1)
Supplement: Supplementary file 1 — Supplementary Information(PDF 11138 kb) [file 41467_2018_3613_MOESM1_ESM.pdf]

The oldest magnetic record in our Solar System identified using nanometric imaging and numerical modeling

Shah et al.

Supplementary Information

Supplementary Note 1

Micromagnetic Modeling of a Large Grain

The temperature-dependent electron holography observations presented in this article were recorded from a kamacite grain of approximate size 458 x 98 x 60 nm with a near-uniform magnetization that remains stable up to at least 500°C.

In order to understand the magnetic domain state of this grain and to determine its paleomagnetic stability, we conducted numerical micromagnetic simulations, which define both the possible magnetic domain states that are compatible with the observations and its stability, which is determined by magnetic energy barrier to other possible local energy minimum domain (LEM) states.

The micromagnetic finite-element model builds the geometry of the magnetic grain from a large number of tetrahedral elements, with the magnetization defined in terms of a constant length vector at each node of each element. Stable micromagnetic states were then found by optimizing the free magnetic energy calculated from the magnetostatic, crystalline and exchange forces within the grain. The material constants that scale each of these forces were taken to be those for pure Fe at 300°C. The chosen magnetic constants reflect the chemical composition of the grains that was determined by EDS analysis to be near-pure metallic Fe. A temperature of 300°C was chosen because we are interested in magnetic domain states and their thermal stability at the maximum temperature to which these magnetic grains are thought to have been exposed since the time of their formation approximately 4.6 billion years ago[1].

Supplementary Note 2

Local Energy Minimum Domain States

The range of possible magnetic domain states was first examined by performing 1000 separate magnetic domain state calculations, each for a different random initial state. The magnetic domain state energies, which can be seen in Supplementary Figure 4, exhibit an expected log-normal distribution[2]. In any thermomagnetic process during which a grain cools slowly from above its Curie point, it is likely that it will find a low-energy state. For the purpose of this study, it was necessary to find a magnetic domain state with both a low

magnetic energy and a domain state that was consistent with the experimental electron holographic magnetic induction maps.

For example, two possible magnetic domain states with different energies are shown in Supplementary Figure 5. The domain state in Supplementary Figure 5a has an energy of 1.6502 x 10^{-16} Joules and comes from near the peak of the domain state energy distribution. The simple 3-vortex state implied by the corresponding simulated 2-dimensional magnetic induction map disguises the true nature of its 3-dimensional magnetization state, which is considerably more complex. Domain state (b) has the lowest energy of all of the domain states found, with a value of 1.52462 x 10^{-16} Joules. Although it has a much simpler domain state, the simulated magnetic induction map is not consistent with experimental observations shown in Figure 2 performed using electron holography.

Two further domain states, which were examined in detail, are shown in Supplementary Figure 6. Both states produce simulated magnetic induction maps that are consistent with our observations and both have energies near the minimum of the energy distribution. Despite the superficial similarity of these domain states, the low helicity regions (colored green) are oriented in opposite directions, with domain states LEM3 and LEM4 having a normalized x-component of the magnetization (aligned with the grain elongation) of 0.76 and -0.75, respectively.

Supplementary Note 3

Thermal Stability of Domain states

Due to the similarity of the simulated magnetic induction maps for LEM3 and LEM4 to those observed experimentally, the NEB method with a convergence constraint of minimum action[3,4] was used to determine the minimum energy path and thus the energy barrier between two such domain states. In this method, a series of domain states was constructed along an initial guess of the path and these domain states were then optimized so that they were on the optimal minimum energy path.

The result of NEB optimization between domain states LEM3 and LEM4 is shown in Supplementary Figure 7. A highly complex path is observed, compared to those reported in this study for much smaller grains. The path is characterized by a multitude of intermediate minima, but has a maximum energy barrier along the path of 4.64 x 10^{-17} Joules. At a temperature of 300°C, for which these domain states were calculated, Néel's equation (See Methods) yields a relaxation time that is of many orders of magnitude greater than the age of the Solar System.

However, the multitude of intermediate minima along the energy path indicates that magnetic domain transitions with much smaller energy barriers may be present. One such candidate for the lowest energy barrier is 11% along the minimum energy path. This domain state was extracted from the minimum energy path, optimized to ensure it is a true local energy minimum state and given the name LEM_p11 (see Supplementary Figure 8a), before recalculating the new NEB minimum energy path. The result is shown in Supplementary Figure 8b.

A comparison between the start and end states (Supplementary Figure 6a and 7a) for this energy path shows very similar domain states with almost identical normalized magnetizations along the x axis of +0.76). The most significant difference in domain state is the location of the small vortex in the top part of the grain. Despite this small difference, the energy barrier to this domain transition produces an extremely long relaxation time at 300°C of at least 3.74×10^{174} billion years.

There is always a possibility that other LEM states may provide lower energy barriers. One other transition was found for a perturbation of the vortex core in the lower part of the grain (LEM_p55), as shown in Supplementary Figure 9a. The minimum energy path between LEM3 (Supplementary Figure 6a) and LEM_p55 (Supplementary Figure 9a) again shows an extremely high stability, with a minimum relaxation time of 3.59×10^{8} billion years.

It is surprising that small perturbations of the domain state in such kamacite grains are associated with such high stabilities. The perturbation of the magnetization affects only a small part of the grain, over a maximum length of approximately 50 nm. Domain stabilities of 50 nm-sized Fe grains have much lower magnetic stability. For example, a 50 nm diameter sphere of Fe has a relaxation time of only 112 microseconds. The reason for the hugely increased stability in the case of the kamacite grain examined in this study is that, even though the change in magnetization is small, the magnetic coupling to the rest of the magnetization state in the grain hugely increases the resistance to a domain state change.

Although our numerical studies cannot claim to be exhaustive, they support the experimental observations that are reported in the main section of this article and suggest that multi-vortex pseudo-single-domain magnetization states are highly stable and more than capable of retaining a magnetic recording for timescales that are far in excess of the age of the Solar System.

Supplementary Figures

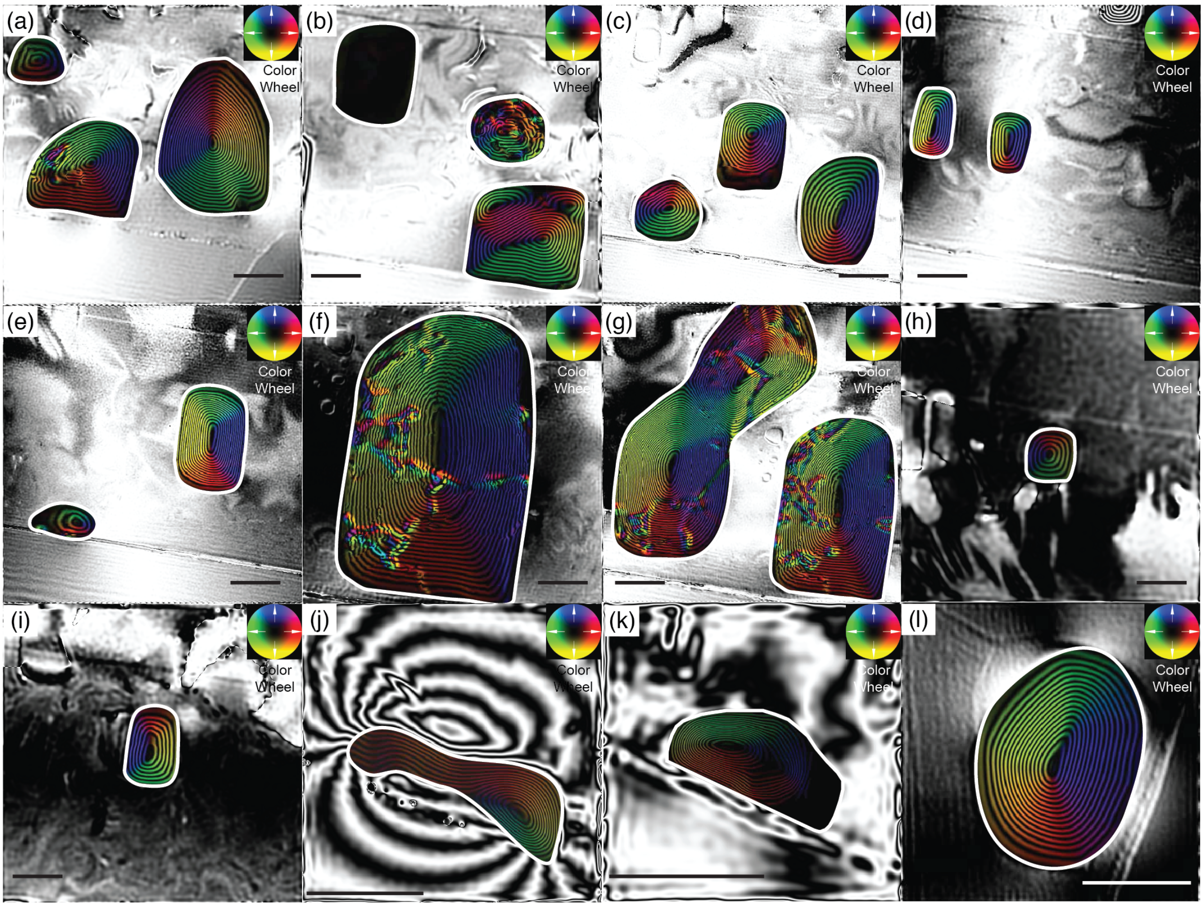

Supplementary Figure 1 – Visualizing the magnetization of kamacite grains in Bishunpur dusty olivine – (a-l) Magnetic induction maps of kamacite grains in dusty olivine reconstructed from electron holograms recorded at room temperature. The contour spacing is π radians. The direction of the projected in-plane magnetic induction is indicated by the color wheel. One of the grains in (b) did not reverse its magnetization direction in the TEM, and a magnetic induction map could not be produced. The defects to the magnetic induction maps for the grains in (a), (f), and (g) are phase reconstruction artefacts due to diffraction contrast (typically a result of grain thickness variations) present in the recorded electron holograms. Scale bars represent 200 nm.

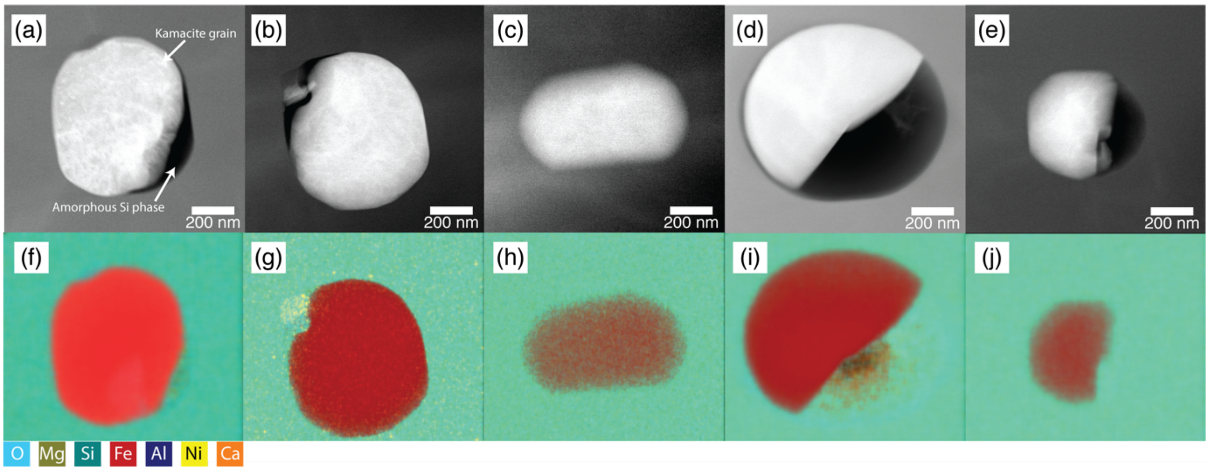

Supplementary Figure 2 – Chemical analysis of Bishunpur dusty olivine – (a-e) High angular annular dark field (HAADF) images of Bishunpur dusty olivine (kamacite grains in forsterite matrix) recorded at the Ernst Ruska-Centre for Microscopy and Spectroscopy with Electrons, Forschungszentrum Jülich, using an FEI Titan 80-300 (S)TEM equipped with a spherical aberration corrector and operated at 300 kV. (f-j) Chemical analysis performed using energy dispersive X-ray spectroscopy (EDS) with a spatial resolution of 2 nm. The elements displayed in the color map are labelled below (f), indicating that the kamacite grains are almost pure Fe, with minor (<< 1%) Ni. The reduction process that precipitated the Fe metal also produces amorphous silica visible as the dark regions next to the bright Fe grains in the HAADF images (a-e).

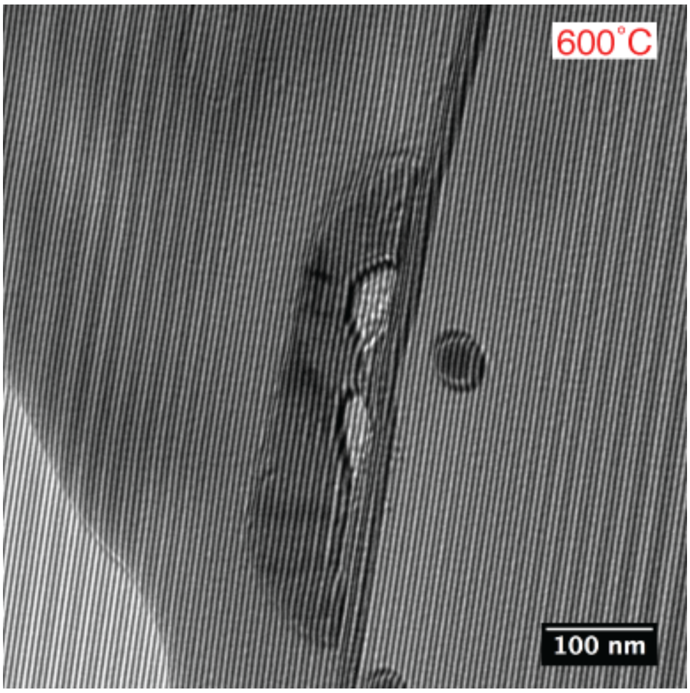

Supplementary Figure 3 – Chemical alteration observed during *in-situ* heating – Electron hologram of the grain observed during *in-situ* thermal demagnetization. The hologram was acquired at 600˚C in magnetic-field-free conditions using a Lorentz lens as the imaging lens rather than the conventional microscope objective lens. Interference fringes are associated with the electron biprism being operated at 50 V to perform phase data acquisition.

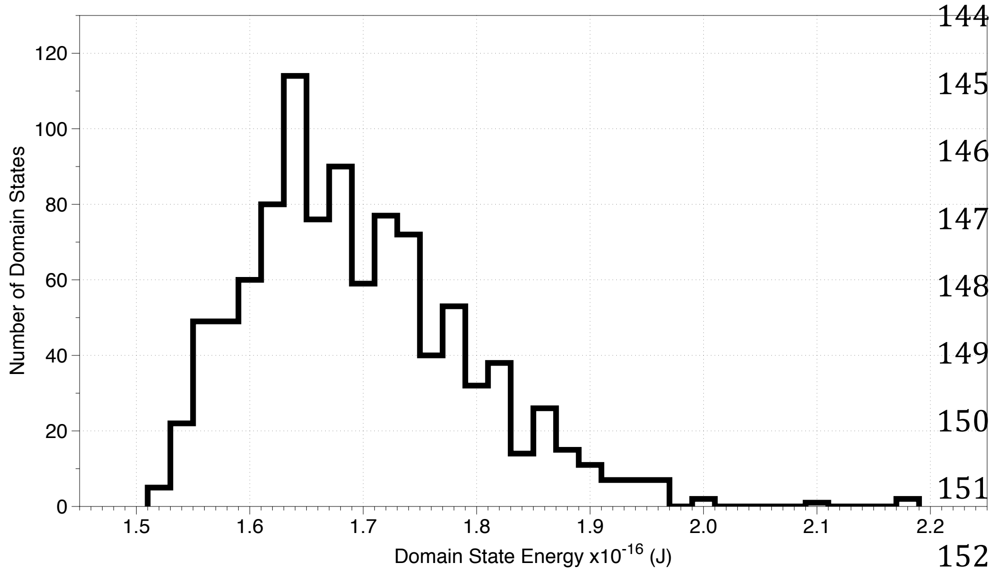

Supplementary Figure 4 – Calculated domain state energy distribution for the large grain – Distribution of domain state energies found for 1000 random initial guesses for the kamacite grain of approximate size 458 x 98 x 60 nm modelled after the grain observed for the temperature-dependent electron holography experiment.

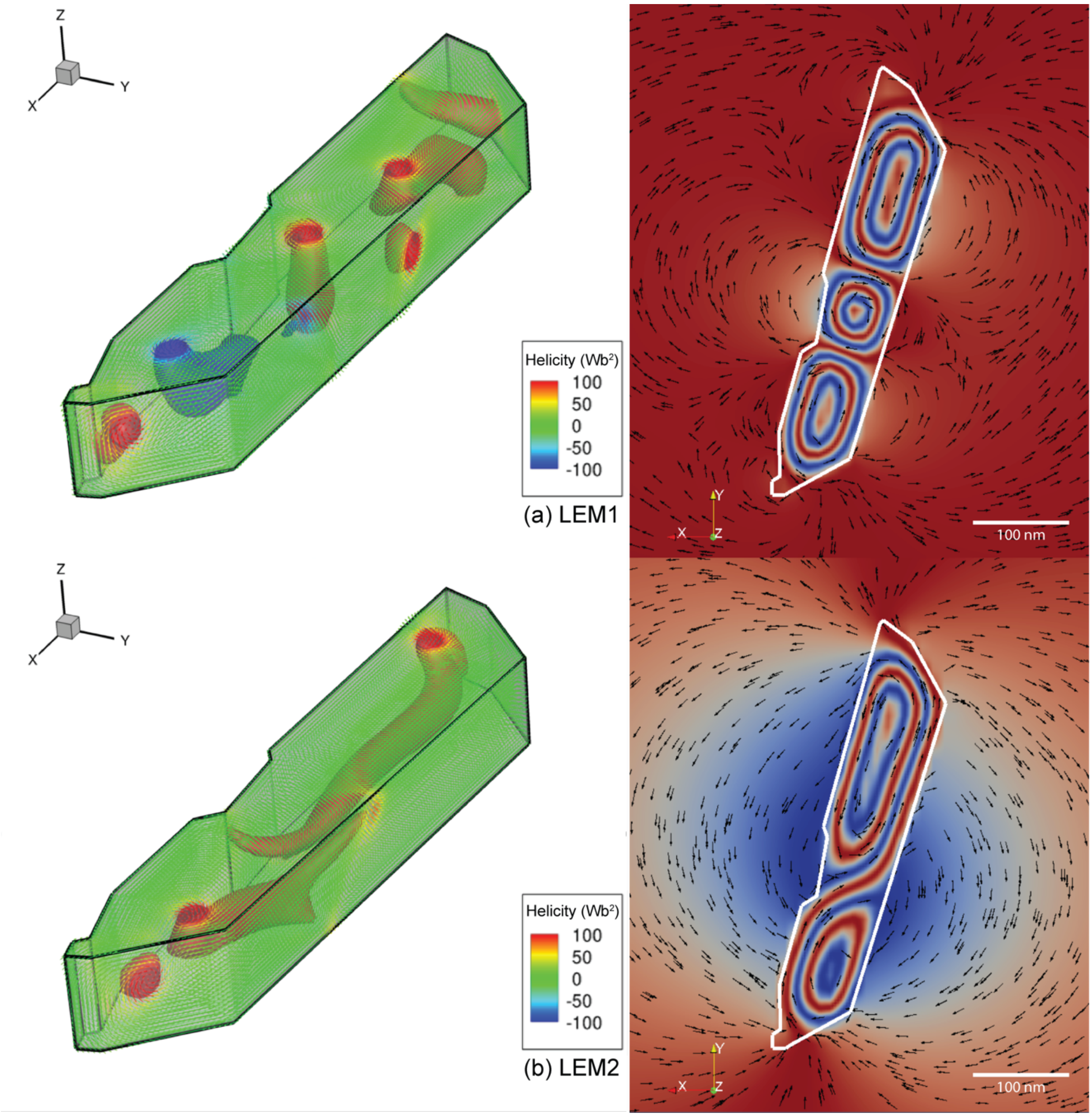

Supplementary Figure 5 – Large grain magnetic domain states LEM1 and LEM2 – (a, b) Magnetic domain states LEM1 and LEM2 corresponding to different parts of the log-normal distribution of domain state energies shown in Supplementary Figure 4. The images on the left are the respective magnetization states. The magnetization vectors have been colored according to the local value of the helicity. Within each grain, an iso-surface has been drawn at helicity values of ±100 Wb^2, which show the presence of vortex cores of opposite polarity. The images on the right are corresponding the simulated electron holographic magnetic induction maps. Domain state (a) occurs near the peak of the energy distribution of Supplementary Figure 4, while state (b) is the lowest energy state found.

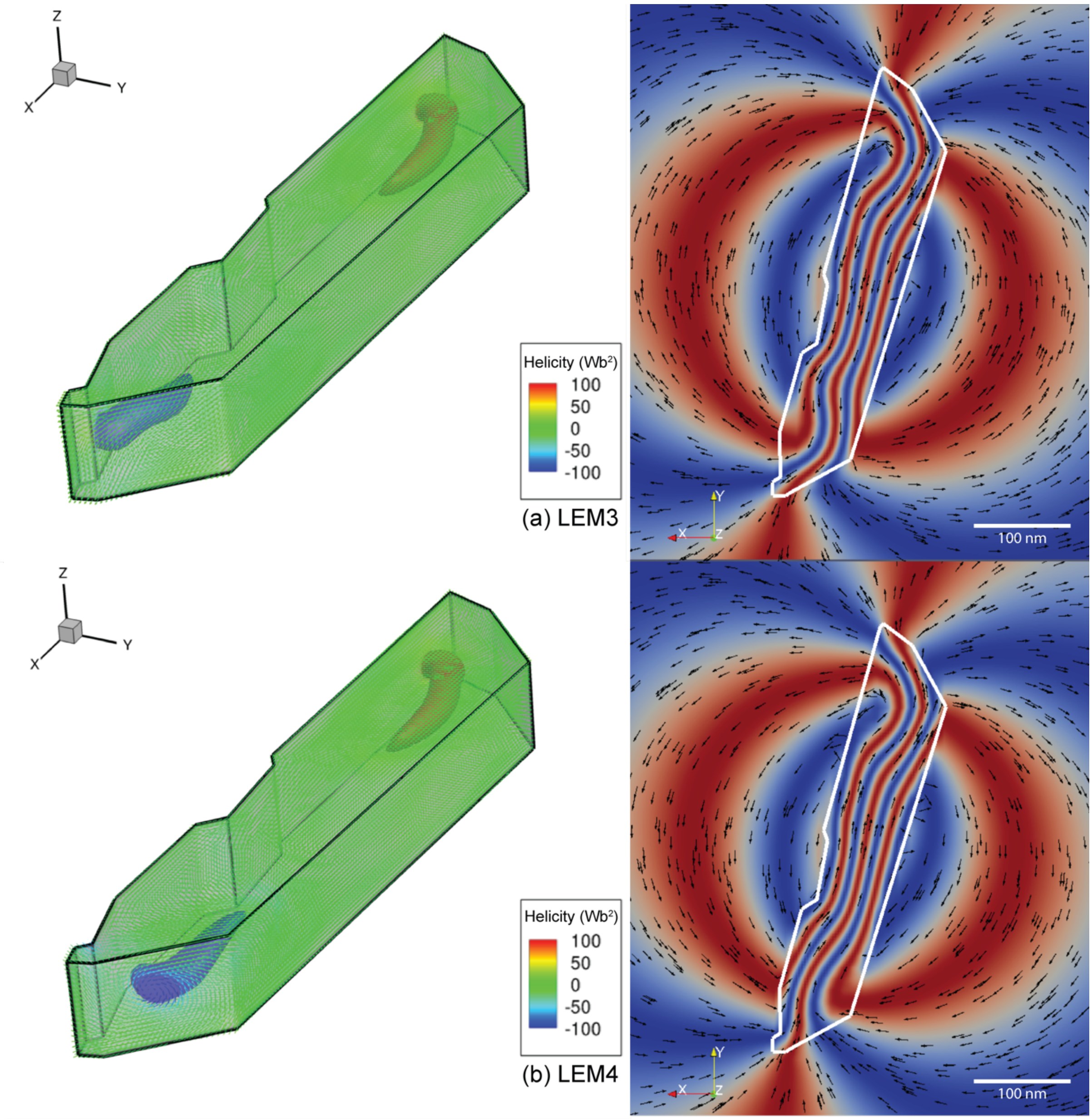

Supplementary Figure 6 – Large grain magnetic domain states LEM3 and LEM4 – (a, b) Magnetic domain states LEM 3 and LEM4 and corresponding electron holographic magnetic induction maps similar to those observed experimentally, with energies of (a) 1.5405 x 10^{-16} J and (b) 1.5383x10^{-16} J.

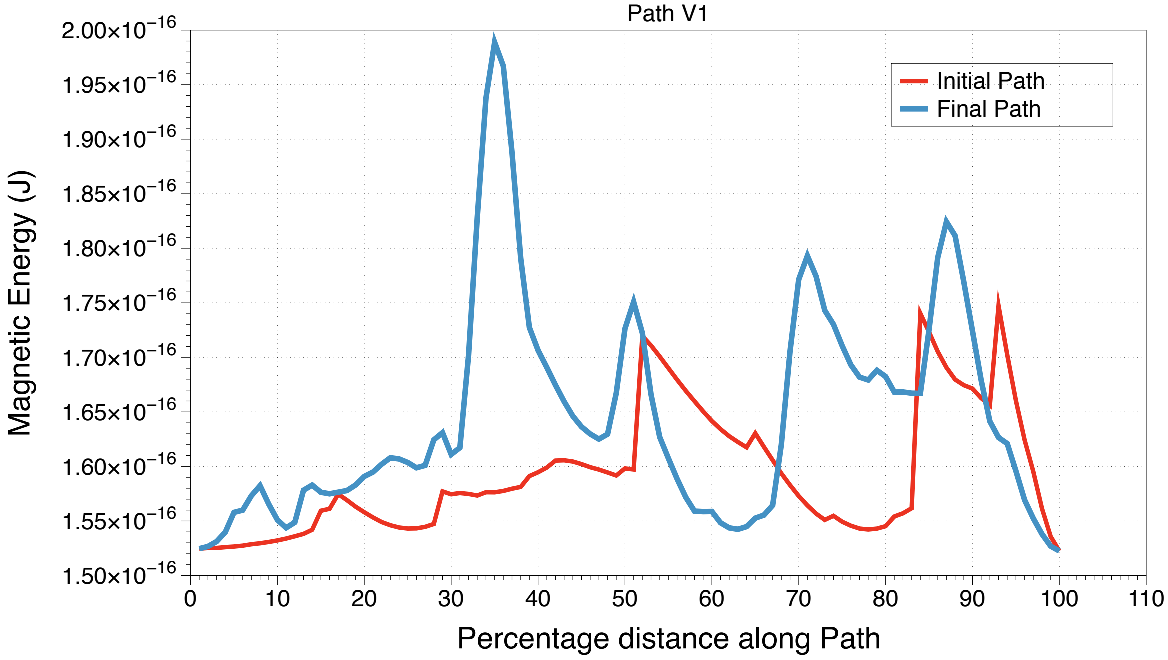

Supplementary Figure 7 – Magnetic transition path between LEM3 and LEM4 – Initial guess and optimised minimum energy path between states LEM3 and LEM4.

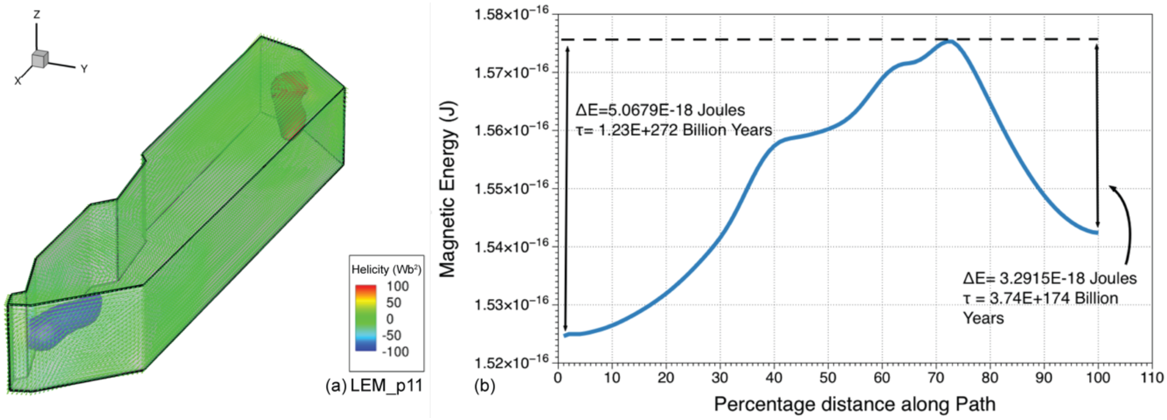

Supplementary Figure 8 – Large grain magnetic domain state LEM_p11 and transition path between LEM3 and LEM_p11 – (a) End LEM state (LEM_p11) used for the NEB minimum energy path between LEM3 and LEM_p11 shown in (b).

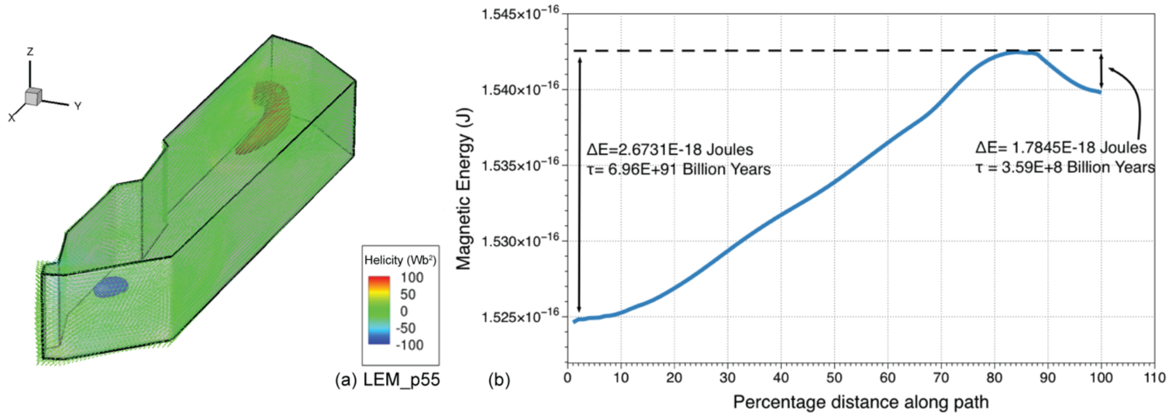

Supplementary Figure 9 – Large grain magnetic domain state LEM_p55 and transition path between LEM3 and LEM_p55 – (a) End LEM state (LEM_p55) used for the NEB minimum energy path between LEM3 and LEM_p55 shown in (b).

Supplementary References

1. Rambaldi, E. R. & Wasson, J. T. Metal and associated phases in Bishunpur, a highly unequilibrated ordinary chondrite. *Geochim. Cosmochim. Acta* **45,** 1001–1015 (1981).
2. Hagen, C. W. & Griessen, R. Distribution of activation energies for thermally activated flux motion in high-Tcsuperconductors: An inversion scheme. *Phys. Rev. Lett.* **62,** 2857–2860 (1989).
3. Fabian, K. & Shcherbakov, V. P. Energy barriers in three-dimensional micromagnetic models and the physics of thermo-viscous magnetization in multidomain particles. *arXiv preprint* 1702.00070. (2017).
4. Berkov, D. V. Numerical calculation of the energy barrier distribution in disordered many-particle systems: the path integral method. *J. Magn. Magn. Mater.* **186,** 199–213 (1998).
