# Supplementary material for: The oldest magnetic record in our solar system identified using nanometric imaging and numerical modeling
Source: Nat Commun. 2018 Mar 21;9:1173. doi: 10.1038/s41467-018-03613-1 (PMC5862876; doi:10.1038/s41467-018-03613-1)
Supplement: Supplementary file 3 — Description of Additional Supplementary Files(PDF 171 kb) [file 41467_2018_3613_MOESM3_ESM.pdf]

Description of Additional Supplementary Files

File Name: Supplementary Movie 1
Description: Path between two local energy minimum magnetization states. Video of the path between two local energy minimum magnetization states for a cuboid with an equivalent volume to a cube of 35 nm diameter extended to an axial ratio of 1.5. The initial and final states are non-uniform vortices through the short sides of the Fe cuboid. The overlaid graph shows that the energy barrier for this transition is 390 kBT, which corresponds to a relaxation time that is much greater than the age of the Solar System at room temperature.
